# Monozygotic twins and triplets discordant for amyotrophic lateral sclerosis display differential methylation and gene expression

**DOI:** 10.1038/s41598-019-44765-4

**Published:** 2019-06-04

**Authors:** Ingrid S. Tarr, Emily P. McCann, Beben Benyamin, Timothy J. Peters, Natalie A. Twine, Katharine Y. Zhang, Qiongyi Zhao, Zong-Hong Zhang, Dominic B. Rowe, Garth A. Nicholson, Denis Bauer, Susan J. Clark, Ian P. Blair, Kelly L. Williams

**Affiliations:** 1 Centre for Motor Neuron Disease Research, Faculty of Medicine and Health Sciences, Macquarie University, Sydney, New South Wales Australia; 2 Australian Centre for Precision Health, University of South Australia Cancer Research Institute, School of Health Sciences, University of South Australia, Adelaide, Australia; 3 South Australian Health and Medical Research Institute, Adelaide, South Australia Australia; 4 Institute for Molecular Bioscience, University of Queensland, Brisbane, QLD Australia; 5 Epigenetics Research Laboratory, Genomics and Epigenetics Division, Garvan Institute of Medical Research, Sydney, New South Wales Australia; 6 Health and Biosecurity Business Unit, Commonwealth Scientific and Industrial Research Organisation, Sydney, New South Wales Australia; 7 Queensland Brain Institute, University of Queensland, Queensland, Australia; 8 Department of Clinical Medicine, Faculty of Medicine and Health Sciences, Macquarie University, Sydney, New South Wales Australia; 9 ANZAC Research Institute, University of Sydney, Sydney, New South Wales Australia; 10 Molecular Medicine Laboratory, Concord Hospital, Sydney, New South Wales Australia; 11 St Vincent’s Clinical School, UNSW Sydney, New South Wales, Australia

**Keywords:** DNA methylation, Gene expression, Methylation analysis, Gene expression analysis, Amyotrophic lateral sclerosis

## Abstract

Amyotrophic lateral sclerosis (ALS) is a fatal neurodegenerative disease characterised by the loss of upper and lower motor neurons. ALS exhibits high phenotypic variability including age and site of onset, and disease duration. To uncover epigenetic and transcriptomic factors that may modify an ALS phenotype, we used a cohort of Australian monozygotic twins (n = 3 pairs) and triplets (n = 1 set) that are discordant for ALS and represent sporadic ALS and the two most common types of familial ALS, linked to *C9orf72* and *SOD1*. Illumina Infinium HumanMethylation450K BeadChip, EpiTYPER and RNA-Seq analyses in these ALS-discordant twins/triplets and control twins (n = 2 pairs), implicated genes with consistent longitudinal differential DNA methylation and/or gene expression. Two identified genes, *RAD9B* and *C8orf46*, showed significant differential methylation in an extended cohort of >1000 ALS cases and controls. Combined longitudinal methylation-transcription analysis within a single twin set implicated *CCNF*, *DPP6*, *RAMP3*, and *CCS*, which have been previously associated with ALS. Longitudinal transcriptome data showed an 8-fold enrichment of immune function genes and under-representation of transcription and protein modification genes in ALS. Examination of these changes in a large Australian sporadic ALS cohort suggest a broader role in ALS. Furthermore, we observe that increased methylation age is a signature of ALS in older patients.

## Introduction

Amyotrophic lateral sclerosis (ALS) is a fatal neurodegenerative disease characterised by the rapidly progressive loss of the upper and lower motor neurons. Disease onset commonly occurs in middle to late age^1^ and typically results in death within three to five years. Existing treatments are of limited effect, and despite intensive effort, the pathogenic mechanisms underlying disease are still poorly understood. A recognised family history (familial ALS, FALS) is seen in approximately 10% of cases while the remainder are considered sporadic (SALS)^2^. The familial and sporadic forms of the disease are clinically and pathologically indistinguishable^3^. To date, the only proven cause of ALS are gene mutations leading to motor neuron death. Pathogenic repeat expansions in the *C9orf72* gene and missense mutations in the *SOD1* gene are the most frequent known causes of ALS worldwide, yet no cause has been identified for the majority of patients (>80%^2^). Even in those individuals with a proven causal gene mutation, inter- and intra-familial phenotypic heterogeneity is commonly observed^1,4^. Age of disease onset may vary by more than 60 years and disease duration may be measured in months or in decades. Affected individuals, particularly those with a *C9orf72* repeat expansion, may present with ALS or frontotemporal dementia (FTD), or a mixed phenotype. Causal mutations may show incomplete penetrance^4^ and indeed monozygotic twins are more commonly discordant for ALS than concordant^5^. Taken together, this phenotypic variability suggests a significant contribution from modifying factors in disease manifestation.

Epigenetic and transcriptional profiling have implicated differential DNA methylation and/or gene expression in ALS. *C9orf72* has been shown to have increased methylation^6,7^ and decreased transcription^8,9^ in ALS/FTD patients with the pathogenic repeat expansion. Other major ALS genes, however, including *SOD1*, *FUS* and *TARDBP*, are generally unmethylated and show no differences between patients and controls^10–12^. Nevertheless, changes in expression of some ALS genes is apparent in sporadic disease^13^. Whole methylome and transcriptome studies in spinal cord and blood tissue have found global changes^10,14,15^ and implicated various genes, pathways and several overlapping themes including changes that affect immune response^14,16,17^ and cellular transport^18,19^.

Disease discordant monozygotic (MZ) twins hold great potential for studies that seek to identify epigenetic and transcriptomic factors that modify the phenotype of complex human diseases. Identical twin studies can account for confounding factors such as genetic variation and the early development environment. Such studies have informed understanding of phenotypic variation in Parkinson’s disease^20^, Alzheimer’s disease^21^, systemic lupus erythematosus^22^, and depression^23^, among others. Previous DNA methylation studies of known causal ALS genes in ALS-discordant MZ twins found no aberrant methylation between twins^24,25^, while twin-based methylome-wide studies suggested a different epigenetic age in affected twins^25,26^ and identified potentially altered GABA signalling^25^ and immune response^27^. Nevertheless, further studies are required because the differentially methylated sites implicated in initial screens have often failed to be validated in targeted studies using bisulphite pyrosequencing^26^. Similarly, candidate molecular pathways have shown limited overlap between twin sets^25^ and changes in methylation are yet to be linked to changes in transcription. It remains unclear which of the observed differences in either DNA methylation or gene expression reflect ALS discordance between co-twins. It is also unclear whether these differences in DNA methylation correlate with differential gene expression on a transcriptome-wide scale.

In this study, we undertook comprehensive methylome- and transcriptome-wide analysis of a longitudinal ALS-discordant cohort comprising MZ triplets and twins, representing the three most common types of ALS: *C9orf72*-linked ALS, *SOD1*-linked ALS and sporadic ALS. We analysed methylome- and transcriptome-wide data, independently and in combination, in an attempt to identify disease-relevant methylation changes and their downstream impact. Co-twin analyses indicated a significant interaction effect between age and disease status on DNA methylation age, with older twins showing a consistent difference between ALS-affected and unaffected co-twins in a longitudinal series. Furthermore, we identified several genes likely to contribute to ALS through integration of longitudinal twin genome-wide DNA methylation and transcription data, further assessed in a large sporadic ALS case-control cohort.

## Results

### ALS-discordant and control twin/triplet sets

**Figure 1:**
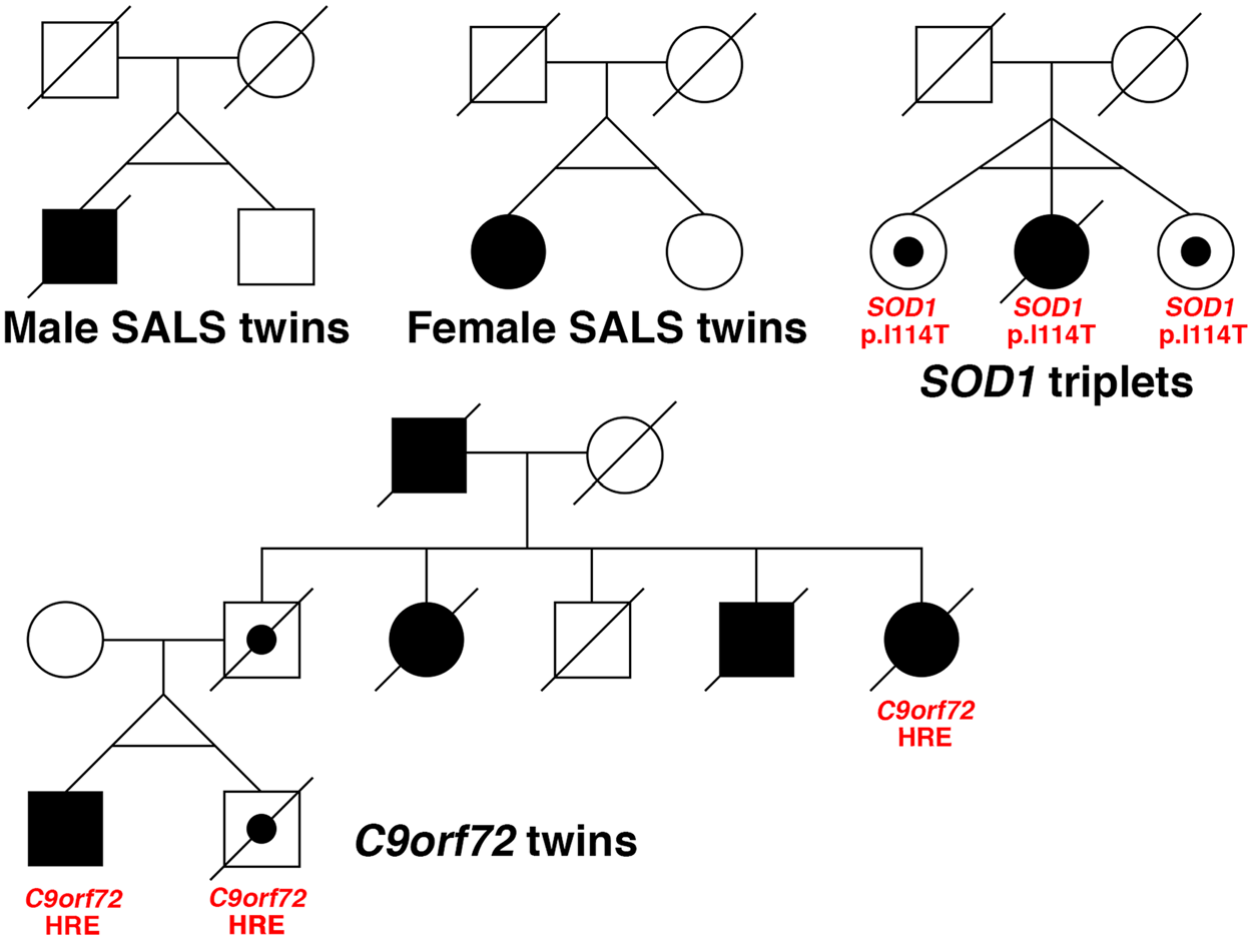
ALS-discordant twin/triplet set pedigrees. Pedigrees for four sets of ALS-discordant twins/triplets, with gene mutations indicated. Circles represent females and squares represent males. Diagonal lines indicate deceased individuals. Filled shapes indicate ALS, open shapes with a dot indicate mutation carriers and open shapes are unaffected non-carriers. Horizontal lines between twins/triplets indicate confirmed monozygosity. HRE: hexanucleotide repeat expansion.

Clinical and sample information for the three discordant MZ twin sets, one discordant MZ triplet set and two control twin sets are included in Table 1. Pedigrees and extended pedigrees are shown in Fig. 1. All individuals with ALS have been screened for causal mutations in known ALS genes. The FALS twin set has a pathogenic hexanucleotide repeat expansion in *C9orf72*. The FALS triplet set harbours a *SOD1* p.I114T mutation.

**Table 1:** Twin cohort details.

MZ set	ALS	Disease Status	Sex	Mutation	Age of onset	Age at sampling^A^	Disease duration (months)	450K samples (n)^B^	RNA-Seq samples (n)	EpiTYPER samples (n)
Female SALS twin set	SALS	ALS	F		42.7	43.5–45.1	Alive at 51 months	7(+1)	—	—
		Unaffected	F			43.9–45.1		7(+1)	—	—
Male SALS twin set	SALS	ALS	M		78.5	79.8–80.2	28.4	3(+1)	3	—
		Unaffected	M			79.8–80.2		3(+1)	3	—
*C9orf72* twin set	FALS	ALS	M	*C9orf72* HRE	52	54.1	36	1	—	1
		Asymptomatic	M	*C9orf72* HRE		54.3–55		2	—	2
*SOD1* triplet set	FALS	ALS	F	*SOD1* p.I114T	50	50.3	Unknown	1	—	1
		Asymptomatic	F	*SOD1* p.I114T		50.3		1	—	1
		Asymptomatic	F	*SOD1* p.I114T		50.3		1	—	1
Control twin set 1	NA	Control	F		NA	46.1	NA	1	—	
		Control	F			46.1		1	—	
Control twin set 2	NA	Control	M		NA	36.8	NA	—	—	1
		Control	M			31.8–43.0^C^		—	—	3^C^

HRE: hexanucleotide repeat expansion; FALS: familial ALS; SALS: sporadic ALS; ^A^Presence of an age range indicates longitudinal samples were collected; ^B^Number of technical replicates during blood collection indicated in brackets; ^C^Middle sample matched to co-twin.

### Targeted analysis of methylation in mutation-known MZ sets

To assess whether differential methylation of the *C9orf72* or *SOD1* CpG islands were associated with the disease discordance we observe in the *C9orf72* twin set and *SOD1* triplets, we investigated the status of CpG methylation of the *C9orf72* and *SOD1* CpG islands. To perform a high-density, targeted analysis, we used EpiTYPER, with additional support from a number of Infinium HumanMethylation450K CpG sites present in the same region.

#### *SOD1* methylation in the *SOD1* MZ triplet set shows a consistent methylation pattern

**Figure 2:**
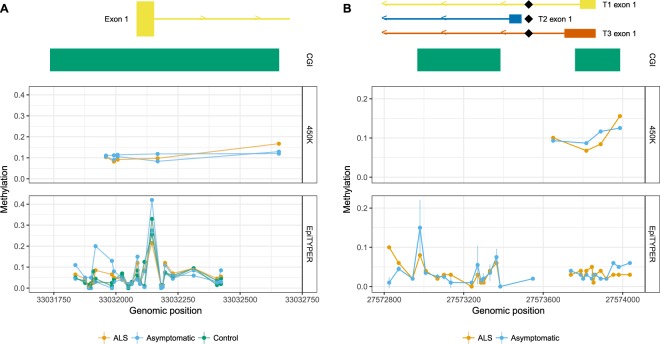
Neither *SOD1* nor *C9orf72* **CpG islands are differentially methylated between mutation-positive ALS-discordant twins/triplets.** The relative location of targeted CpG islands (CGI) and exon 1 are indicated for *SOD1* (**A**, top) and *C9orf72* (**B**, top). (**A**) Methylation of the CpG island spanning the promoter region and exon 1 of *SOD1* does not show differential methylation between an ALS-affected triplet and unaffected co-triplets, concordant for *SOD1* p.I114T. Methylation status was determined using both EpiTYPER (bottom) and 450K (middle) assays. (**B**) Transcript variants (T1, T2, and T3) and the position of the repeat expansion (black diamond) relative to exon 1 are shown for *C9orf72* (top). Methylation of the *C9orf72* promoter region/expansion flanking CpG islands are not differentially methylated between ALS-discordant co-twins that carry the *C9orf72* hexanucleotide repeat expansion in either EpiTYPER (bottom) or 450K data sets (middle).

We used EpiTYPER to quantify methylation of the upstream *SOD1* CpG island encompassing the *SOD1* promoter region and exon 1 in the discordant MZ triplets carrying the *SOD1* p.I114T mutation and a pair of control twins from another *SOD1* p.I114T family that were negative for the *SOD1* mutation. Additionally, five *SOD1* CpG sites present in the Infinium HumanMethylation450K data set were located within the CpG island (Fig. 2A). Neither the 23 CpG units within the CpG island, nor the five 450K *SOD1* CpG sites, showed any consistent methylation differences between ALS affected and ALS unaffected MZ triplets, nor control twins (Fig. 2A).

#### No differences were observed in *C9orf72* methylation in the *C9orf72* MZ twin set

The quantitative methylation status of two CpG islands associated with *C9orf72* was determined using EpiTYPER. The amplicons covered the entirety of both CpG islands, the promoter region and adjacent intronic/intergenic regions. The intronic pathogenic (GGGGCC)_n_ repeat expansion (indicated with a black diamond in Fig. 2B) is flanked by the two CpG islands. In the disease discordant FALS twin set harbouring a *C9orf72* expansion, methylation across the GpG island (CGI) measured by the EpiTYPER assay are highly concordant and generally unmethylated (Fig. 2B). Similarly, in the four 450K probes associated with *C9orf72*, none of the CpG sites show a clear difference in methylation between the co-twins (Fig. 2B).

### Whole methylome analysis of disease discordant MZ twins/triplets

#### Co-twin/triplet differences in DNA methylation age (DNAm) reflects an age-dependant effect

**Figure 3:**
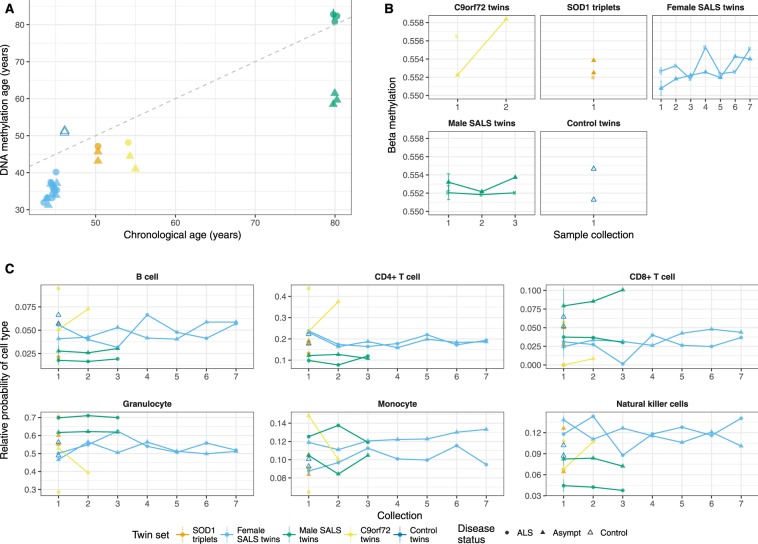
DNA methylation age (DNAm), but neither global mean methylation nor cell composition, varies between ALS-discordant twins/triplets. (**A**) DNA methylation age was more discrepant between ALS discordant twins with increasing chronological age (p = 1.3E-5), with greater DNAm aging in affected twins/triplets than their unaffected co-twin/-triplets, when controlling for age and sex. (**B**) Global mean methylation across 386183 CpG sites found no significant difference in global methylation between ALS-affected and unaffected co-twins/-triplets when controlling for age and sex (p = 0.08). (**C**) Proportions of six white blood cell types over time were estimated for ALS-affected and unaffected twins/triplets. Proportions for each cell type were not significantly associated with disease status of twin/triplet samples when controlling for age and sex (CD4+ T cells, p = 0.77; CD8+ T cells, p = 0.24; Monocytes, p = 0.60; B cells, p = 0.21; Natural killer cells, p = 0.52; granulocytes, p = 0.63). For collection times with technical replicate samples per person, points represent the mean at that time, with the standard deviation indicated with a line.

Horvath’s DNA methylation age algorithm^28^ used methylation levels of 353 CpG sites to predict the epigenetic age from each twin/triplet sample in Table 1. DNA methylation age has a high correlation with chronological age across multiple tissue types, thus enabling calculation of the biological age of an individual. We tested the association of methylation age with disease status and chronological age in a mixed model while controlling for sex. The effect of disease status on methylation age was found to be highly dependent upon chronological age (p = 1.3E-5, Fig. 3A). Briefly, with increasing age, asymptomatic co-twins were estimated to have a younger epigenetic age than their ALS-affected twin. This result was most evident in the approximately 20-year difference in methylation age between twins in the oldest disease discordant twin set of this cohort (Fig. 3A).

#### Global methylation and cell type proportions do not show any effect of disease status

Global methylation was calculated as the mean methylation across all Infinium HumanMethylation450K CpG sites passing data processing (n = 386183). No significant effect of disease on global methylation was found when controlling for sex and age at sample collection (p = 0.08, Fig. 3B). To better reflect influence on transcription, CpG sites were classified according to CpG density: high density CpG islands, intermediate density in islands, island shores, and low CpG density. Mean methylation within each of these four levels of CpG density does not show any effect of disease status (HC, p = 0.93; IC, p = 0.99; ICshore, p = 0.82; LC, p = 0.093, Supplementary Fig. S2). Full blood count data were unavailable from twin/triplet sets at time of sample collection. Therefore, blood cell proportions for each twin/triplet sample were estimated from methylation data using Houseman *et al*.’s algorithm^29^ and the six cell types were assessed for association with disease status. Disease status did not have a significant effect on any of the cell types when controlling for age at sample collection and sex (all p > 0.2, Fig. 3C).

#### Differentially methylated probes were identified across discordant MZ twins/triplets

**Figure 4:**
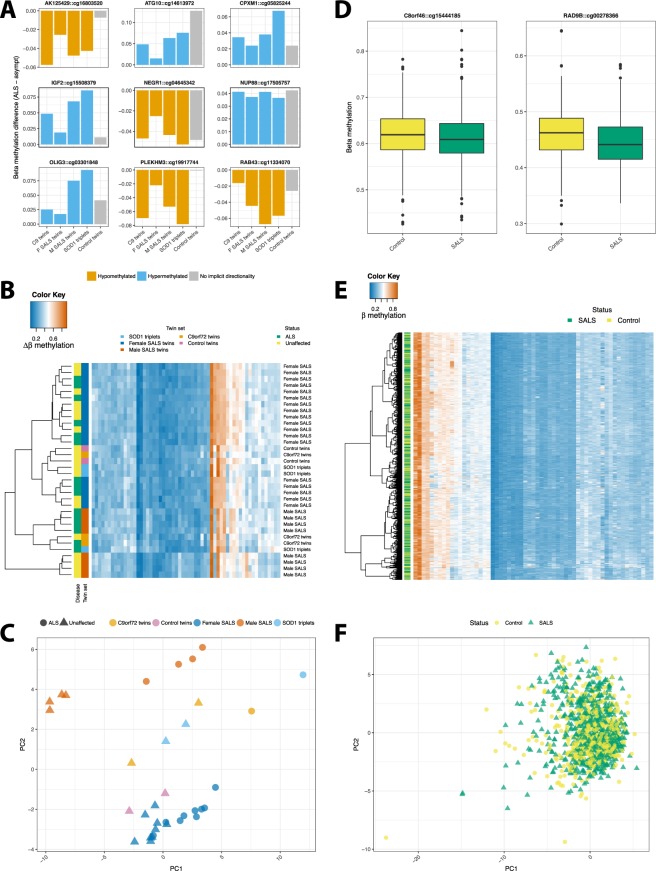
Top identified DMPs do not cluster by disease in MZ and case-control cohorts. (**A**) Of 59 DMPs found across all discordant twin sets, these 9 were top ranked for the combination of statistical significantly differences between affected and unaffected co-twins/-triplets and the magnitude of differences across twin/triplet sets. Per twin/triplet set differences are shown, with the ALS-affected sibling as the reference for direction of methylation. Gene annotation and CpG name are indicated as *gene::cpg*. Bar colour indicates hypomethylation of the ALS-affected twin (orange) or hypermethylation of the affected twin/triplet (blue) relative to their unaffected co-twin/-triplets. Grey bars represent the magnitude of difference in methylation in the control twin set and thus have no implicit directionality. They are shown with the same directionality as the discordant twins to facilitate comparison of the magnitude of the difference in methylation. (**B**) The 59 DMPs identified across discordant twin/triplet sets were used to cluster the samples in the MZ cohort (n = 34). Overall, methylation was similar across samples for most DMPs, and samples did not cluster by disease, nor perfectly by twin/triplet set. (**C**) Principal Components Analysis (PCA) across discordant twin/triplet sets and the control twin set using the same 59 DMPs also showed that samples did not cluster by disease, but approximately by individual for those where longitudinal samples were available. (**D**) Of the 59 DMPs identified across discordant twin/triplet sets, two were significantly different between cases (SALS) and controls in a large extended cohort (n SALS = 646, n controls = 533). Both cg15444185, annotated to *C8orf46*, and cg00278366, annotated to *RAD9B*, were hypomethylated in SALS samples (cg15444185, *β* = −0.06, adjusted p = 0.049; cg00278366, *β* = −0.0771, adjusted p = 2.5E-5) when controlling for age and sex. (**E**) The top 59 DMPs identified across all discordant twin set do not cluster by disease status in a sporadic case control cohort. (**F**) PCA also demonstrates that these top 59 twin DMPs do not cluster by disease status in a sporadic case control cohort.

The established DNA methylation twin-study method of statistical significance and the magnitude of pairwise methylation differences were combined to detect differentially methylated probes in discordant MZ twin studies. 59 probes were identified as differentially methylated across twin/triplet sets (full list in Supplementary Table S5, 9 top-ranked probes shown in Fig. 4A). All 59 probes were used for hierarchical clustering and principal components analysis (PCA) of the longitudinal MZ cohort to investigate the presence of a disease signature. Both hierarchical clustering and PCA did not indicate that samples cluster by disease status, but rather approximately by twin set and individual, where longitudinal samples were available (Fig. 4B,C). The 59 probes were subsequently investigated in our large case-control 450K methylation extended data set (n = 646 SALS cases and 533 unrelated controls). After FDR correction, 2 of the 59 probes showed significantly differential methylation between cases and controls when controlling for age and sex (*RAD9B*, cg00278366, p = 2.5E-5; *C8orf46*, cg15444185, p = 0.049, Fig. 4D; full results for all 59 CpGs in Supplementary Table S6). As observed in the MZ cohort, hierarchical clustering and PCA of this probe list in the case-control cohort does not indicate any power to discriminate between ALS and control samples (Fig. 4E,F), nor sex or age group.

#### Differentially methylated probes (DMPs) identified within discordant MZ twin/triplet sets implicates new genes and existing ALS genes

**Figure 5:**
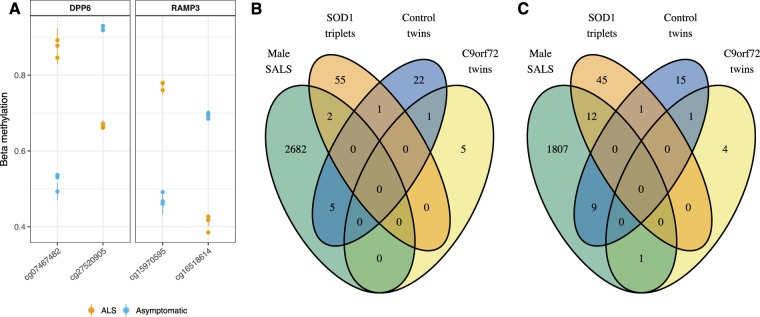
Most differentially methylated probes (DMPs) per twin set were unique to one twin set including known ALS genes. DMPs within a twin/triplet set were those with a difference in *β*-methylation ≥0.25. (**A**) Within the male SALS twin set, two probes were identified which annotated to *DPP6*, and two additional probes annotated to *RAMP3*. Multiple data points per person at each probe indicate longitudinal sampling. For collection times with technical replicate samples per person, points represent the mean at that time, with the standard deviation indicated with a line. (**B**,**C**) Generally, DMPs were unique to a twin set, while no differences in methylation (>0.25) were detected in the female SALS twins. (**B**) The number of DMPs within a twin set varied from <10 in *C9orf72* twins to >2500 male SALS twins (Supplementary Fig. S3B,D). Only two of these DMPs were found in multiple discordant twin sets. Each of the male SALS twins, *SOD1* triplets and *C9orf72* twins showed overlap with the control twins. (**C**) Within each of the three discordant twin sets and the control twin set DMP lists, multiple probes annotated to the same gene. When comparing these genes rather than individual probes, more shared genes were identified between discordant sets, with 13 genes containing a probe considered differentially methylated in multiple discordant twin/triplet sets.

Given the clinical heterogeneity in our twin/triplet cohort, within-twin-set differential methylation was also investigated. Using a threshold of a difference in *β*-methylation ≥0.25 between co-twins or the affected triplet and the mean of the unaffected triplets, we identified 0 DMPs in female SALS twins, 6 in C9orf72 twins, 58 in SOD1 triplets, 2689 in male SALS, and 29 in control twins (Supplementary Fig. S3A–E). Up to 11 probes were annotated per gene in the male SALS twin list of DMPs, for a total of 1829 genes identified. The 506 genes to which multiple male SALS twin probes annotate are given in Supplementary Table S7, which includes two genes previously associated with ALS, *DPP6* (Dipeptidyl Peptidase Like 6) and *RAMP3* (Receptor Activity Modifying Protein 3) (Fig. 5A). No other discordant twin/triplet set had multiple probes annotated to the same gene. Across all discordant twin/triplet sets, 2 probes (Fig. 5B) and 13 genes (*BDKRB2*, *CHRD*, *DYSF*, *HOXD11*, *IRX4*, *ISL1*, *JOSD1*, *mir*_*44*, *NKX2-5*, *NXN*, *OTX1*, *POU4F2*, *RFX4*, Fig. 5C) were identified in multiple twin/triplet sets. None of these probes or genes were also identified in the control twin set. Each of the male SALS twins’ DMPs, *C9orf72* twins’ DMPs and *SOD1* triplets’ DMPs showed minimal overlap with the control twins DMPs (5, 1, and 1 DMPs, respectively, Fig. 5B, Supplementary Table S8). Similarly, minimal overlapping genes-annotated-to-DMPs were identified between the control and discordant twins/triplets, with 9, 1, and 1 genes respectively (Fig. 5C, Supplementary Table S8).

### Transcriptome-wide analysis of disease discordant MZ siblings

#### Differentially expressed genes within male SALS twins implicates immune function and cell signalling functional pathways in sporadic ALS

**Figure 6:**
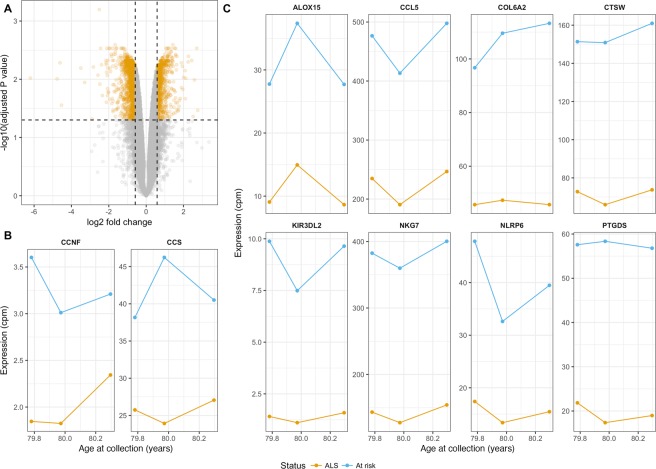
Genes that showed consistent longitudinal differential expression in SALS twins included known ALS genes. (**A**) Seven hundred and fifty genes were identified as differentially expressed with a minimum fold change of 1.5 (vertical lines) and significant FDR-corrected p-value (horizontal line) in the male SALS twins. (**B**) Expression of two previously reported ALS genes, *CCNF* and *CCS*, identified as differentially expressed in male SALS twins. Gene expression is shown for all three collections in each twin. (**C**) Expression of the top 8 genes (as ranked by limma) are shown for all three longitudinal collections of the male SALS twins.

**Figure 7:**
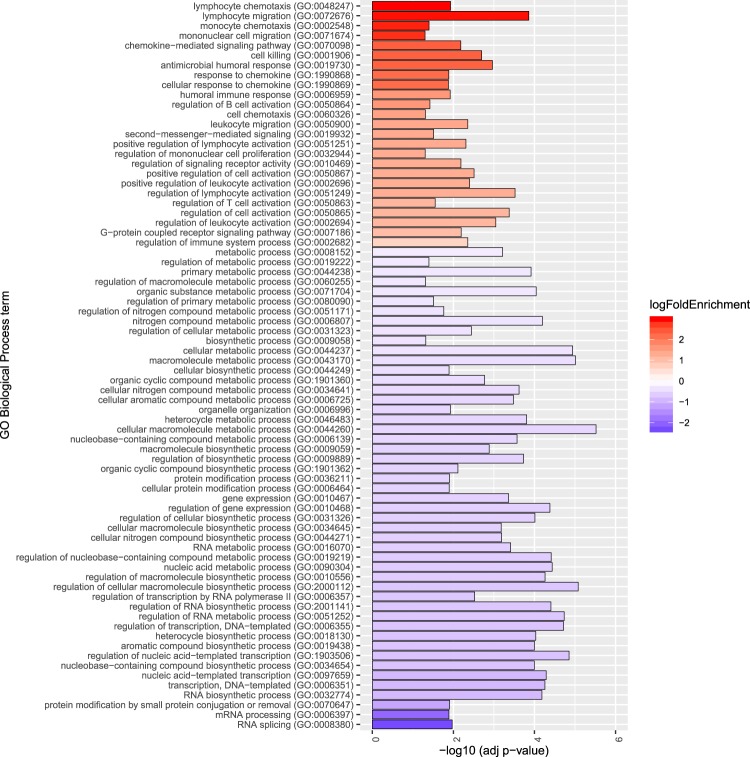
Significantly enriched Gene Ontology (GO) terms implicate enrichment of immune function in the ALS co-twin. GO analysis of the 750 longitudinally differentially expressed genes from the male SALS twins identified 74 significantly enriched biological processes or pathways, shown on the y-axis of the graph. Adjusted p-value (using FDR method) is indicated by the height of the columns on the graph (x-axis). Log2 fold enrichment (logFoldEnrichment) of a GO biological process is indicated by depth of colour, and direction of gene representation (red = over-representation in affected co-twin, blue = under-representation in affected co-twin. Results demonstrate over-representation of genes associated with immune function and cell signalling, and under-representation of genes largely related to transcription and protein modification.

Using limma voom to detect genes differentially expressed between male SALS twins while controlling for repeated sampling, we identified 4179 genes as significant following FDR correction (p < 0.05). Of these, 750 genes also had a fold change of 1.5 or greater (Fig. 6A, top genes shown in Fig. 6C, full list in Supplementary Table S9). Notably, *CCNF* and *CCS*, both known ALS genes, were identified as significantly downregulated in the ALS twin compared to their unaffected co-twin (*CCNF*: logFC = 0.70, t = 3.99, FDR = 0.027; *CCS*: logFC = 0.70, t = 6.42, FDR = 0.008, Fig. 6B). Gene Ontology (GO) analysis of these 750 genes identified 74 terms significantly enriched in this list. Over-representation of genes was seen in 25 terms associated with immune function and cell signalling, while there was an under-representation of genes associated with 45 terms, largely related to transcription and protein modification (Fig. 7, Supplementary Table S10).

#### Validation of twin differentially expressed genes in a case-control cohort

**Figure 8:**
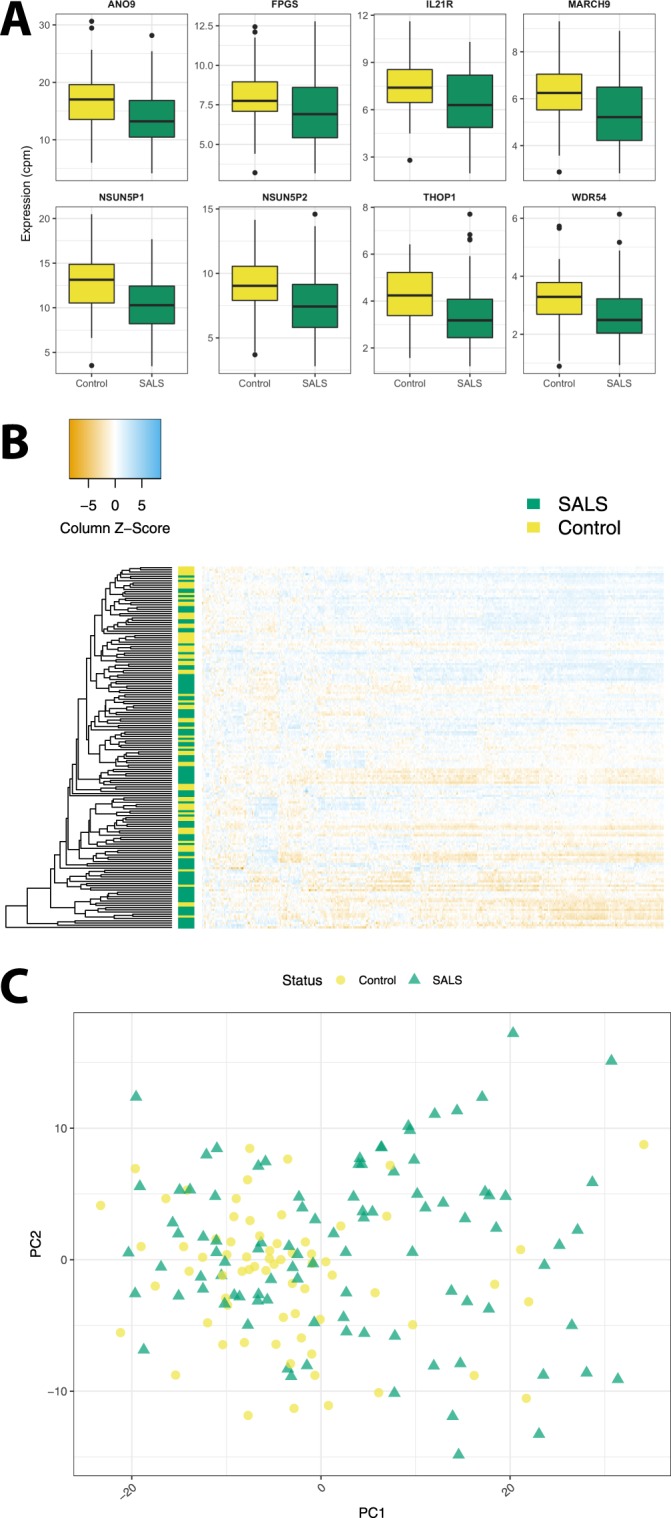
DEGs identified in male SALS twin are validated in a case-control cohort. Of the 750 DEGs identified in the male SALS twins, only 379 genes were present in the sporadic case-control cohort. (**A**) Two hundred and thirteen of these were validated as differentially expressed between SALS (n = 96) and controls (n = 69) when controlling for sex (Supplementary Table S7) and the top 8 are shown here. (**B**) Hierarchical clustering of the sporadic ALS and control cohort by these 379 genes did not identify disease-based clusters. (**C**) Similarly, principal Components Analysis (PCA) of the sporadic ALS and control cohort by these 379 genes did not identify disease-based clusters.

Within the validation RNAseq data set of SALS and controls, 379 of the 750 genes identified in the male SALS twins were present. When analysed with limma while controlling for sex, 213 of the 379 genes were differentially expressed between cases and controls, yet none also showed a minimum fold change of 1.5 (top 8 of 213 genes shown in Fig. 8A, 213 genes in Supplementary Table S11). *CCNF* was not present in the case-control data set, while *CCS* was not validated (log FC = 0.13, t = 1.99, FDR = 0.075). Hierarchical clustering and PCA of the 379 genes did not identify clusters representing disease status (Fig. 8B,C).

### Integration of genome-wide methylation and transcriptome data sets identifies 12 genes both differentially methylated and differentially expressed

**Figure 9:**
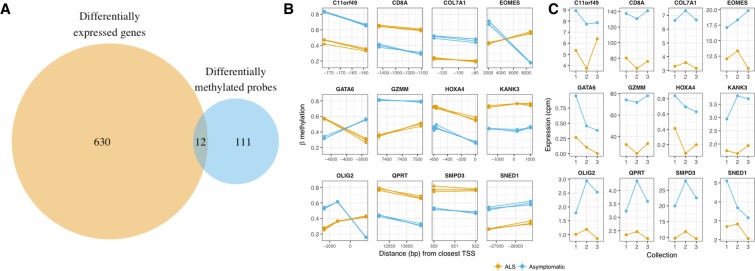
Twelve overlapping genes were identified in the male SALS twins DMPs and DEGs. (**A**) While 506 genes were identified as having multiple probes with a difference in *β*-methylation (≥0.25) between the ALS-discordant male SALS twins, only 123 of these genes were present in the matching RNA-Seq data. 642 of 750 genes identified as differentially expressed were present in the matching DNA methylation data. Twelve of these genes were both differentially expressed and differentially methylated (*C11orf49*, *CD8A*, *COL7A1*, *EOMES*, *GATA6*, *GZMM*, *HOXA4*, *KANK3*, *OLIG2*, *QPRT*, *SMPD3*, *SNED1*). (**B**) On the HumanMethylation 450K beadchip, multiple CpG probes are annotated to each gene. Methylation of all probes annotated to each of the twelve differentially expressed and differentially methylated genes shows strong consistency within each co-twin. Distance from the transcription start site in base pairs is shown on the x axis. Multiple data points per person at each probe indicate longitudinal sampling. Duplicate collections within a time point are shown as the mean with the standard deviation indicated by a line. (**C**) Longitudinal expression of the 12 shared genes, (*C11orf49*, *CD8A*, *COL7A1*, *EOMES*, *GATA6*, *GZMM*, *HOXA4*, *KANK3*, *OLIG2*, *QPRT*, *SMPD3*, *SNED1*), is consistently different between ALS discordant male SALS co-twins over time.

To increase the likelihood of detecting biologically meaningful disease-related alterations, RNA-Seq and Infinium HumanMethylation450K data sets were combined for the male SALS twins. Of 506 genes having at least one differentially methylated CpG probe annotated to them in the male SALS twins, 123 are also identified in the entire post-processing RNA-Seq data set of 13718 genes. Conversely, of the 750 genes present in our top DEG list, 642 also have at least one CpG probe mapped to the same gene in the full post-processing 450K set of 24073 genes. When comparing the gene lists of the 123 differentially methylated genes and the 642 differentially expressed genes from the same twin set, 12 genes (*C11orf49*, *CD8A*, *COL7A1*, *EOMES*, *GATA6*, *GZMM*, *HOXA4*, *KANK3*, *OLIG2*, *QPRT*, *SMPD3*, *SNED1*) were present in both gene lists (Fig. 9A–C).

## Discussion

Using a longitudinal cohort of MZ twins and triplets that are discordant for ALS, we have conducted both a targeted and genome-wide DNA methylation study in conjunction with a sample-matched transcriptomic study. Our cohort is representative of the clinical heterogeneity (age of disease onset, disease duration) frequently observed in ALS cohorts. We have shown that DNA methylation age is the most consistently altered epigenetic signature in ALS. In addition, we observed a higher frequency of unique peripheral blood methylation changes within twin/triplet sets compared to shared methylation changes across twin/triplet sets. However, combined analysis of peripheral blood methylation and transcription detected ALS-relevant changes. These data suggest that the epigenetic and transcriptomic landscape of ALS may be highly complex with numerous small perturbations and various pathways, only some of which are common, contributing to disease.

Epigenetic age was significantly associated with disease in an age-dependent manner, such that affected twins/triplets have an older DNA methylation age than their unaffected co-twins/-triplets while no such effect was observed in young discordant twins. A clear and consistent difference was apparent between the oldest twins in the study, and to a lesser extent, within both middle-aged twin/triplet sets. This pattern of increased methylation age in ALS affected twins is consistent with previous studies of *SOD1*, *C9orf72* and SALS disease-discordant twins^25,26,30^. Increased DNA methylation age has been linked to increased mortality^31^ and age has been shown to be a major risk and prognostic factor for ALS^32^. Our results also reflect a contribution of ageing to disease risk. Methylation age has also been previously linked to age of onset in ALS patients with a *C9orf72* repeat expansion^30^, while we observed a similar phenomenon in our sporadic ALS twin sets, with a much greater between-co-twin difference in DNA methylation age in our late onset twin set compared to our early onset twin set. Further investigation in extended ALS cohorts, specifically mutation-known FALS and SALS would be worthwhile to confirm the contribution of increased DNA methylation age to ALS.

When assessing genome-wide DNA methylation using a magnitude and statistical ranking method, we identified 59 probes differentially methylated in all ALS twins/triplets compared to their unaffected co-twin/-triplets. These 59 probes were selected from high CpG density regions of the genome, therefore considered biologically relevant as they are more likely to affect gene expression. Annotation of the probes to the closest gene transcription site and subsequent gene ontology analysis implicated developmental processes. However, clustering of these 59 DMPs were unable to discriminate between affected and healthy twins, or sporadic cases and controls. Yet, two of these probes were confirmed as significantly differentially methylated in the case-control analysis. *C8orf46*’s *Xenopus* homolog gene *vexin* is involved in neurogenesis and highly expressed in the brain^33^, while *RAD9B* responds to DNA damage by moving to the nucleus and contributes to control of the cell cycle^34^. There is a growing body of evidence that DNA damage response is a significant factor in ALS^35^.

We conducted within-twin/triplet-set comparisons to show the *SOD1* triplet set, *C9orf72* twin set and the male SALS twin set each have a moderate number of probes with large differences in methylation (6, 58, and 2689 probes respectively with |Δ*β*| ≥ 0.25). In contrast, the female SALS twin set showed highly consistent methylation across all >386,000 probes (max |Δ*β*| = 0.11). Following annotation of these probes to genes, limited overlap of differentially methylated genes was observed between disease discordant twin sets, consistent with other genome-wide methylation studies in ALS-discordant SALS twin sets^25,27^. This may be reflective of the heterogenous nature of sporadic ALS, or the different methodologies and strategies used to determine differential methylation. A meta-analysis incorporating these studies would be worthwhile to give a broader picture of the methylation signature of sporadic ALS.

It is noteworthy that the four twin sets used in this study represent two distinct genetic forms of disease (*SOD1* and *C9orf72*), along with two cases at extreme age-of-disease-onset ends of the clinical spectrum of sporadic ALS, suggesting again that there may be various epigenetic pathways impacting the phenotype. Some proportion of the observed differences unique to a twin set may result from epigenetic drift^36^, especially as the greatest number of unique differentially methylated probes was identified in the oldest twin set and the least in the youngest twin set. It is therefore likely that disease, as well as age, is contributing to the differential methylation observed. The metabolism and nutritional status of an individual with ALS, compared to their unaffected co-twin, may also influence methylation status^37,38^, and therefore potentially contribute to the differential methylation observed. However, detailed dietary data is not available for the individuals included in this study and therefore could not be assessed. Nevertheless, we identified multiple differentially methylated probes annotated to two genes previously associated with ALS, *DPP6*^39^ and *RAMP3*^40^ in our oldest twin set, the male SALS twins. *DPP6* was the first gene to be associated with sporadic ALS^41^. It has roles regulating dendritic excitability, with membrane hyperexcitability observed in ALS^42,43^. It has also been associated with multiple sclerosis^44^ and spinal muscular atrophy^45^, and as such is worthy of further investigation in broader ALS.

Analysis of transcriptome-wide gene expression in a subset of our disease discordant MZ cohort, the male SALS twins, found 750 differentially expressed genes. 379 of these genes were assessed in our validation sporadic case-control cohort, and 213 were confirmed to be significantly differentially expressed in sporadic ALS. Gene Ontology analysis implicated primarily upregulation of the immune system, which has been previously identified as dysregulated in ALS^14,16,17,27^. Interestingly, *CCNF* and *CCS* were downregulated in the ALS-affected twin. *CCNF* has been identified as a causal ALS and FTD gene in several international cohorts^46^. While transient overexpression has been shown to have deleterious effects in *CCNF* zebrafish models^47^, this is the first report of altered *CCNF* mRNA expression in ALS. *CCS* has been previously linked to *SOD1* in its implication in ALS^48^. Little is known about the effects of altered expression of *CCS* in ALS, but its overexpression in the G93A-*SOD1* ALS mouse model has been linked to accelerated neurological deficits and worsened mitochondrial pathology^49^. It is interesting that we observed lower expression in the ALS twin than their unaffected co-twin, given that overexpression has been linked to disease in both genes. It was an unfortunate limitation of this study that neither gene featured in our post-processing HumanMethylation450K dataset, and that so few of the genes identified had data available in our case-control data set. As such, it would be worthwhile to further investigate disease-dependent expression of the remaining 371 genes.

Comparison of transcriptional and DNA methylation changes in ALS-discordant twin/triplet set(s) indicated that despite many genes being present in only one data set, there was overlap between the two datasets. Of the 750 differentially expressed genes identified in the male SALS twins, 642 had methylation data available, while of the 506 genes to which multiple of the 1366 differentially methylated probes annotated, only 123 were also represented in our gene expression data. When we compared these 642 expression-derived genes and 123 methylation-derived genes, we identified twelve genes: *C11orf49*, *CD8A*, *COL7A1*, *EOMES*, *GATA6*, *GZMM*, *HOXA4*, *KANK3*, *OLIG2*, *QPRT*, *SMPD3*, *SNED1*. Notably, the ALS genes identified from DMPs in the male SALS twin set, *RAMP3* and *DPP6*, were not present in the post-processing male SALS twins RNA-Seq data set. *C8orf46* and *RAD9B* were identified across all twin sets to have a single probe differentially methylated, which was confirmed in our sporadic case-control cohort, however, neither gene was present in our RNA-Seq data set. While *CCNF* and *CCS* were differentially expressed in the male SALS twins, neither gene was present in the methylation dataset. While none of the twelve genes have previously been directly linked to ALS, some indirect links exist. *COL7A1*, as part of the collagen gene family, is related to *COL6A1*, which has been linked to neurodegeneration through impaired autophagy and induction of apoptosis^50^. Additionally, collagen has also been identified as a significant gene ontology term in analysis of DNA methylation in sporadic ALS^11^. *GZMM*, granzyme M, is 1 of 4 gene products from the granzyme family. Granzymes A and B are elevated in ALS serum, with granzyme B correlated to ALS severity^51^. Granzyme B has been further implicated in inducing apoptosis in human ALS motor neurons^52^. *SMPD3*, neutral Sphingomyelinase II, is associated with apoptosis and cell cycle regulation, which have been previously linked to ALS^53,54^. *KANK3* has been suggested as a possible gene contributing to an ALS-linked region on chromosome 17^55^. *QPRT* is involved in the kynurenine pathway, which has been implicated in ALS^56^. These twelve genes, identified when combining DNA methylation and gene expression data, may thus contribute to disease, and warrant further investigation.

Assessment of global methylation and blood cell composition showed no difference between ALS and healthy co-twins. Although a lack of global changes in methylation is consistent with five other sets of ALS-discordant twins^25^, not all studies agree^10,14,15^. It is also interesting that blood cell composition, as determined from whole blood methylation, was not found to vary between affected and unaffected twins, given that upregulation of the immune system and changes in white blood cell populations have previously been demonstrated in ALS^57,58^. This lack of effect in white blood cell estimates may be partly attributable to shared genetic background^59,60^, although a prior study reported differing methylation-derived cell proportion estimates in one ALS-discordant twin pair^27^.

High-density quantitative targeted analysis of the *C9orf72* and *SOD1* gene-associated CpG islands and gene promotors did not identify any differences in methylation status between ALS-discordant MZ twins/triplets carrying mutations in these genes. The general consistency observed in *SOD1* methylation between carriers of *SOD1* mutations suggests DNA methylation of the *SOD1* promoter itself is not likely to be a major mechanism contributing to differences in penetrance in *SOD1*-linked ALS, in line with previous reports^10^. Methylation of *C9orf72* was low in a twin set carrying the *C9orf72* repeat expansion. Methylation of the *C9orf72* promoter and/or the repeat expansion has been reported in the brain and blood of repeat expansion carriers^6,9,61–65^, in some cases with similar low levels of methylation as that observed here. Interestingly, neither of the two prior *C9orf72* twin studies, one ALS concordant and one discordant, detected methylation of *C9orf72*^24,66^, suggesting that *C9orf72* methylation is just one part of the epigenetic story in ALS.

In conclusion, our disease-discordant twin study, utilising longitudinal samples throughout disease progression, demonstrated significant association of DNA methylation age with disease in an age dependent manner. We have also identified an important set of DMPs and DEGs, and associated functional pathways, that may be involved in either ALS pathogenesis or protection from disease. These genes and pathways offer potential targets for future therapeutic treatment for ALS patients.

## Methods

### Participants

The cohort of 1806 total participants used in this study is summarised below. This study was approved by the human research ethics committees of Macquarie University (5201600387) and Sydney South West Area Health Service. Samples from ALS patients, family members, and unrelated controls were obtained from the Macquarie University Neurodegenerative Diseases Biobank, Molecular Medicine Laboratory at Concord Hospital, and the Australian MND DNA bank. Written informed consent was obtained from all study subjects and all methods were performed in accordance with the relevant guidelines and regulations. Most participants were of European descent and patients were clinically diagnosed with definite or probable ALS based on El Escorial criteria^67^. Genomic DNA was extracted from peripheral blood using standard protocols. RNA was extracted from peripheral blood with the QIAsymphony PAXgene blood RNA kit (Qiagen, Hilden, Germany).

#### Twin/triplet cohort

Three ALS discordant monozygotic twin pairs, one ALS discordant MZ triplet set and two control MZ twin pairs were included in this study (Fig. 1 and Table 1). Monozygosity for each twin/triplet set was confirmed using STR fragment analysis and/or SNP microarrays. Longitudinal samples were available from two twin sets (male and female sporadic ALS (SALS) twin sets in Fig. 1). The four discordant twin/triplet sets had previously undergone mutation analysis for known ALS genes and whole genome analysis for novel and/or rare de novo variants.

#### Data processing and extended cohorts

Additional samples were used in this study for data processing (Illumina HumanMethylation 450K, EpiTYPER methylation assays, and RNA-Seq) and for examination of significant findings (Illumina HumanMethylation 450K assay and RNA-Seq). Demographic characteristics between cases and controls in extended cohorts were assessed with t-tests for age and *χ*^2^ tests for sex.

For quality control and processing of the EpiTYPER data, 279 samples with *C9orf72* EpiTYPER data (158 familial ALS/FTD samples, 56 asymptomatic samples (individuals harbouring a causal gene mutation but currently unaffected), and 65 control samples), and 261 samples with *SOD1* EpiTYPER data (123 familial ALS, 65 asymptomatic, and 73 control samples) were used.

For the Infinium HumanMethylation 450K BeadChip, 1658 samples were used in data processing and normalisation. This comprised 889 individuals with sporadic or familial ALS, 92 asymptomatic and 668 controls. The familial ALS and asymptomatic cases largely overlap with the EpiTYPER cohort. The extended cohort subset comprised 650 sporadic ALS individuals and 539 unrelated controls.

One hundred and ninety samples were used for data processing and normalisation of the RNA-Seq data, comprising 114 individuals with ALS (99 sporadic ALS, 15 familial ALS) and 76 unrelated controls. The validation subset comprised of 96 sporadic cases and 69 controls. The majority of the 96 validation sporadic ALS cases were also present in the HumanMethylation 450K BeadChip SALS/control cohort.

### Methylation assays and data processing

All quality control and data processing steps were carried out in R v 3.4.4^68^.

#### EpiTYPER assay

Custom EpiTYPER assays (Sequenom, San Diego, USA) were used to quantify CpG methylation of 56 and 39 CpG units respectively of the two gene-associated CpG islands for *C9orf72* and the gene-associated CpG island upstream of *SOD1*. EpiTYPER uses base-specific cleavage of bisulphite-converted DNA and matrix-assisted laser desorption/ionization time-of-flight mass spectrometry (MADL-TOF MS) to quantify DNA methylation^69^. Primers for overlapping amplicons were designed with Sequenom’s EpiDesigner software to target the CpG island regions, and therefore the promoter regions, as shown in Supplementary Fig. S1. Primer and assay details are available in Supplementary Table S1. Samples were assayed in one or two batches, and either in duplicate or as singletons (Supplementary Table S1). Sample processing was performed by Agena Bioscience (Brisbane, Queensland, Australia). As each gene assay was run across several plates of samples, the highly methylated DNA control was used to calculate the between-plate coefficient of variation, determined to be 4.9% and 2.3% for *SOD1* and *C9orf72* plates, respectively. CpG methylation was quantified as the percentage of methylated cytosines for each CpG unit, where CpG units consist of one or more CpG sites. For units with multiple CpG sites, methylation percentages were normalised by averaging across the number of sites.

#### EpiTYPER data processing

EpiTYPER data processing was adapted from a previously established method^70^. Twin samples were processed together with the full familial cohorts to leverage the increased sample size. In brief, CpG units that failed to meet assay reliability standards were discarded and samples in duplicate were averaged for the remaining CpG units. Given the relatively small number of CpG units remaining after removal of those determined to be unreliable and the relatively high failure rate of samples and units, a two-step sample/unit filtering process was used. First, failed samples, with ≥90% of CpG unit readings missing, were removed, followed by CpG units which were missing data for ≥90% of samples. Second, samples with a low detection success (missing data for ≥15% of units) were removed, and the same threshold applied to remove CpG units with low detection success (units missing data for ≥15% of samples). Finally, any remaining missing values were imputed with the mean for that unit. Following data processing and filtering, 28 of 56 and 23 of 39 CpG units (for *C9orf72* and *SOD1*, respectively) remained for analysis.

#### Infinium Human Methylation 450K v1.2 BeadChip array

Genome-wide methylation was investigated using the Infinium HumanMethylation 450K v1.2 BeadChip (Illumina, San Diego, USA). This microarray provides qualitative methylation values for approximately 480,000 CpG sites distributed throughout the genome. Bisulphite-converted DNA was hybridised to the Infinium HumanMethylation 450K BeadChip. Fluorescence imaging of the BeadChip using an Illumina HiScan SQ scanner successfully generated raw Intensity Data files (.idat) for all samples.

#### 450K data processing

Data processing of the .idat files was adapted from the method presented by^71^. Twin samples were processed together with the full cohort to leverage larger sample sizes. All default settings were used except where otherwise specified. In brief, samples with less than 99% of CpGs detected were removed. shinyMethyl (v. 1.12.0^72^) was used to visually identify possible outliers, with confirmation of sex queries using RnBeads (v. 1.0.0.^73^). Samples with any possibility of incorrect identification were removed. Data were normalised with the dasen function from wateRmelon (v. 1.20.3^74^). Probes that had failed to be detected (threshold p > 0.05) with the minfi (v. 1.22.1^75^) function detectionP were removed (n = 10270 probes). Normalised data were submitted to Horvath’s online DNAm age calculator^28^. Samples that did not strongly correlate (r < 0.85) with the DNAm age results gold standard were removed. Leveraging technical replicate/duplicate samples (n = 30), both 1) in the form of multiple blood collections at the same time and resulting independent DNA extractions (technical replicates) and 2) multiple aliquots of single DNA extractions (duplicates), a custom filtering step was included to identify and remove highly variable probes. Any probe identified to have multiple pairs of technical replicate or duplicate samples with differences greater than three standard deviations from the probe’s mean difference was discarded (n = 38697). Of the remaining probes, any known to cross hybridise, be located on sex chromosomes, or bind to SNPs, were removed (n = 50362)^76^.

Following raw data processing, quantitative CpG methylation values for 1215 samples (including 34 twin samples from Table 1 and 1179 case/control extended cohort samples outlined in Supplementary Table S2) and 386183 probes remained for analysis and examination. Comparison of the case-control extended cohort (Supplementary Table S2) showed that sex (${\chi }_{(1,{\rm{n}}=1179)}^{2}$ = 33.8, p < 0.01) and age (t_1174_ = 4.20, p < 0.01) were significantly different between ALS cases and controls.

### Analysis of methylation data

All statistical analyses were carried out in R v. 3.4.4^68^.

#### Gene-specific targeted methylation analysis of *SOD1* and *C9orf72* in the FALS twin/triplet sets

Methylation of *SOD1* or *C9orf72*, as quantified by both EpiTYPER and 450K assays, was visualised in the relevant monozygotic disease discordant twin/triplets. Four and five 450K CpGs were available in the post processing data set in the targeted region of *C9orf72* (cg05990720, cg11613875, cg14363787, cg23074747) and *SOD1* (cg16086310, cg17253939, cg18126791, cg19948014, cg26893544), respectively. Since only one twin set and one triplet set are available in our cohort for each respective variant, results are descriptive only.

#### Observed differences in DNA methylation age, blood cell composition and global methylation within a twin/triplet set

DNA methylation age was determined from 450K methylation data using the method of Horvath^28^. Blood cell proportions in whole blood derived methylation was estimated from 450K methylation data with the minfi implementation of Houseman *et al*.’s algorithm^29^. Global methylation levels were determined as the mean methylation estimate across all post-processing 450K CpG sites per sample. CpG sites were also divided into one of four categories based on HIL CpG classes (high-density CpG island (HC), intermediate-density CpG island (IC) and non-island (LC); ICshore, intermediate-density CpG island shore that borders HCs)^77,78^ and the mean methylation for each was calculated.

#### Methylome-wide analysis in MZ sets to identify differentially methylated probes

The list of differentially methylated probes (DMP) across all MZ sets was identified using an established ranked magnitude-significance method^79^. In brief, statistical significance per CpG site was determined using a paired t-test on methylation M-values, using the per-patient mean of longitudinal samples and unaffected triplets. The magnitude of the difference in methylation was calculated as the mean difference in *β*-methylation between co-twins. Both methods were used to rank all CpGs, and a final ranked list was determined from the mean of these two ranking methods. Top DMPs were the subset of all CpG probes that met the following two criteria, 1) they were in high CpG density regions of the genome and 2) the ranked list of high-density probes was truncated immediately prior to the first probe to show a difference in the direction of change across the four discordant MZ sets. The ability of these probes to discriminate between ALS and healthy individuals was assessed by hierarchical clustering and principal components analysis of all twin/triplet sets.

Within-twin/triplet set DMPs were also identified. A CpG probe was considered to be differentially methylated within a twin/triplet set where there was an absolute difference in *β*-methylation ≥0.25 between the affected twin and their unaffected co-twin/triplet.

#### Examination of identified twin DMPs in a sporadic ALS cohort

Twin/triplet DMPs were examined in the larger sporadic case-control cohort. Differences between cases and controls for each of the identified probes were analysed, along with the ability of the DMP list to cluster cases and controls separately.

### Gene expression

#### RNA sequencing

Raw sequencing reads in fastq format were generated for male SALS twins (based on longitudinal sample availability) and the sporadic case-control validation cohort as outlined in Supplementary Table S3.

#### RNA-Seq data processing

The quality of raw sequencing reads was evaluated using fastQC (v 0.11.7^80^) for both datasets. Trimming and alignment was performed as outlined in Table 1 using either Trimmomatic (v. 0.36^81^) or Cutadapt (version 1.8.1^82^) and HISAT2 (v2.0.5^83^). All subsequent data processing and analysis was completed in R (v. 3.4.4), using BioConductor packages edgeR (v. 3.18.1^84^) and limma (v. 3.32.10^85^). A standard edgeR TMM normalisation and filtering pipeline was used in data processing, with only those genes where expression was greater than 0.3 counts per million in a minimum of 3 samples (male SALS twins) or 2 counts per million in a minimum of 75 samples (case-control cohort) retained for analysis, which is equivalent to approximately 12–15 raw counts in the smallest library size for each dataset. For the male SALS twins RNA-Seq data, of the 27685 human genes present in the per-gene read counts generated by HTSeq^86^, 13718 genes remained following raw data processing using edgeR^84^. Whereas in the case-control cohort, of the 23368 human genes present in the per-gene read counts generated by HTSeq^86^, 7354 genes remained following raw data processing using edgeR^84^. MDS (multi-dimensional scaling) indicated the presence of three outliers in the case-control cohort, 1 control and 2 SALS samples. All three were removed and final clinical details for the cohort can be found in Supplementary Table S4. Comparison of the RNA-Seq case-control validation cohort (Supplementary Table S4) showed that there were no significant differences in age (t_157.9_ = 1.74, p = 0.08) between the ALS cases and controls, but a difference was observed in sex between cases and controls (${\chi }_{(1,{\rm{n}}=165)}^{2}$ = 6.5, p = 0.01).

#### Differentially expressed genes in MZ twins

To identify differentially expressed genes (DEGs) using the paired longitudinal RNA-Seq samples from the ALS-discordant male SALS twins (Table 1), read count data was analysed using limma^85^, including model terms for longitudinal sample collection and disease status. Voom^87^ transformation was applied prior to modelling. Multiple testing correction using the BH-FDR method^88^ was applied to the full list of post-processing genes.

#### Validation of twin DEGs in a sporadic cohort

Genes identified in twin analyses were investigated for an effect of disease in the full case-control cohort with limma, including sex as a covariate. Data were voom transformed, given the highly variable library sizes. Multiple testing corrections using the BH-FDR method was applied only on the subset of genes identified as differentially expressed in twins. Hierarchical clustering and principal components analysis of the expression of these DEGs in the case-control cohort was assessed.

### Combined methylation and expression analysis

#### Intersect of top CpGs and genes

To identify genes most likely to be altered in disease, results from independent analysis of genome-wide methylation and expression data sets were integrated. Longitudinal RNA-Seq data is only available for one male SALS twin set, therefore we first identified the overlap between top DEGs and the genes annotated to the most differentially methylated probes within that twin set. We extended this analysis by overlapping the same list of DEGs with the genes annotated to the top differentially methylated probes across all combined twin sets.

### Gene Ontology analysis

Gene Ontology enrichment analysis^89,90^ for biological processes was applied to the genes identified as differentially expressed in male SALS twins. The gene list was analysed with PANTHER overrepresentation tests (GO Ontology database release 2018-08-09^91^). Enrichment was tested relative to all genes detected in the appropriate post-processing data set. Fisher’s exact test with FDR correction was used.

### Statistics

All analyses were carried out in R (v. 3.4.4)^68^. Linear mixed effects models were used to analyse DNAm age, blood cell type proportions and global mean M-methylation. Modelling was carried out using the lmer function in the package lme4 (v. 1.1.14^92^) for DNAm age and mean methylation, while a mixed effects beta regression for cell type proportions was applied with the glmmTMB function from the glmmTMB package (v. 0.2.2.0^93^). Blood cell type proportions were increased by 0.001 to all estimates to avoid taking the log of zero. All mixed models assessed the effect of disease status while controlling for age at sample collection and sex. When analysing DNAm age, the interaction of disease and age at collection was also tested. Random effects were introduced for repeated sampling within co-twins, and a random intercept per twin/triplet set. When modelling cell types, due to convergence issues, the random slope for repeated sampling was dropped, leaving random intercepts for each co-twin and twin set. Likelihood ratio tests were used to determine significance of model terms. Linear models were used for case-control examination of probes identified in the MZ cohort, with the same fixed effect terms of age at sample collection and sex as described for mixed models. Hierarchical clustering utilised the package cluster (v. 2.0.6), with Manhattan distance and ward clustering methods for 450K data^94^, and Spearman correlation distance and average linkage clustering for log-transformed RNA-Seq count data^95^. Where appropriate, technical replicates are shown as means with error bars indicating standard deviation (unless otherwise stated).

## Supplementary information

Supplementary Tables S1-S4 and Figures S1-S3

Supplementary Table S5

Supplementary Table S6

Supplementary Table S7

Supplementary Table S8

Supplementary Table S9

Supplementary Table S10

Supplementary Table S11

## Data Availability

The datasets generated and/or analysed during the current study are not publicly available since our ethics permission does not cover sharing of data to third parties but are available from the corresponding author on reasonable request.
