# Supplementary material for: Monozygotic twins and triplets discordant for amyotrophic lateral sclerosis display differential methylation and gene expression
Source: Sci Rep. 2019 Jun 4;9:8254. doi: 10.1038/s41598-019-44765-4 (PMC6547746; doi:10.1038/s41598-019-44765-4)
Supplement: Supplementary file 1 — Supplementary Tables S1-S4 and Figures S1-S3 [file 41598_2019_44765_MOESM1_ESM.pdf]

Supplementary Information

Monozygotic twins and triplets discordant for amyotrophic lateral sclerosis display differential methylation and gene expression

Ingrid S. Tarr, Emily P. McCann, Beben Benyamin, Timothy J. Peters, Natalie A. Twine, Katharine Y. Zhang, Qiongyi Zhao, Zong-Hong Zhang, Dominic B. Rowe, Garth A. Nicholson, Denis Bauer, Susan J. Clark, Ian P. Blair and Kelly L. Williams

Table S1: EpiTYPER assay details

**Gene**	**Amplicon**	**Genomic region**	**Primer sequences**	**Batch 1^**	**Batch 2**
C9orf72	1	chr9:27573165-27573554	F: 5'-ATAAGGGATGGGGATTTGGTTT-3'<br>R: 5'-CAACCTATAACAAACTCTAAAACTCAAAA-3'	Singlicate	Singlicate
	2	chr9:27572780-27573201	F: 3'-ATAAGGGATGGGGATTTGGTTT-5'<br>R: 3'-AATACCCAAATCACAATAATCACTTC-5'	10% duplicate, 90% singlicate	Singlicate
	3	chr9:27573681-27573951	F: 3'-AGGGTGGGAAAAATAAAAATATATATT-5'<br>R: 3'-CTTAAAATAAAAAAACAAAACAAATTACCC-5'	-	Singlicate
	4	chr9:27573945-27574107	F: 3'-GGGTAATTTGTTTTGTTTTTTTATT-5'<br>R: 3'-AAATTTCATCCACCAATTATATATTAAA-5'	-	Singlicate
SOD1	1	chr21:33031765-33032120	F: 5'-TGAAAAGAAGGTTGTTTTTTTTAT-3'<br>R: 5'-TCCCAACCCTTACCTTCTACTC-3'	Duplicate	Singlicate
	2	chr21:33032146-33032453	F: 5'-GAGTAGAAGGTAAGGGTTGGGA-3'<br>R: 5'-CCAAAAACCACAAAAAACAACA-3'	Duplicate	Singlicate

^ *C9orf72* amplicons 3 and 4 were not included in batch 1

Table S2: Post-filtering clinical summary of Infinium HumanMethylation450K case-control validation cohort

**Disease status**	**Number samples**	**Number female (%)**	**Mean age (sd)**	**Mean age (sd) female**	**Mean age (sd) male**
SALS	646	245 (38.3%)	62.9 (12.0)	64.3 (12.0)	62.0 (12.0)
Control	533	295 (55.3%)	59.9 (12.6)	58.6 (12.6)	61.6 (12.4)

Table S3. RNA sequencing and processing summary for longitudinal male SALS twins and case-control cohort

**Cohort**	**Library prep kit**	**Library size (mean)**	**Sequencing platform**	**Read length**	**Average reads per sample**	**Trimming program**	**Alignment program**	**% reads aligned**
Male SALS twins	Illumina TruSeq Stranded mRNA kit LT	314–324bp (320bp)	Illumina NovaSeq 6000	101 bp×2	128.72 million	Trimmomatic (v. 0.36)	HISAT2 (v2.0.5)	83%
Case-control	Illumina TruSeq Stranded mRNA kit HT	200–500bp (350bp)	Illumina HiSeq 2000 (12 samples in multiplex)	126 bp×2	27.62 million	Cutadapt (version 1.8.1)	HISAT2 (v2.0.5)	95%

Table S4. Post-filtering clinical summary of RNA-Seq case-control validation cohort

**Disease status**	**Number samples**	**Number female (%)**	**Mean age (sd)**	**Mean age (sd) female**	**Mean age (sd) male**
SALS	96	38 (39.6%)	63.1 (14.4)	61.2 (14.2)	64.4 (14.5)
Control	69	42 (60.9%)	59.5 (12.3)	57.5 (12.3)	62.6 (11.9)

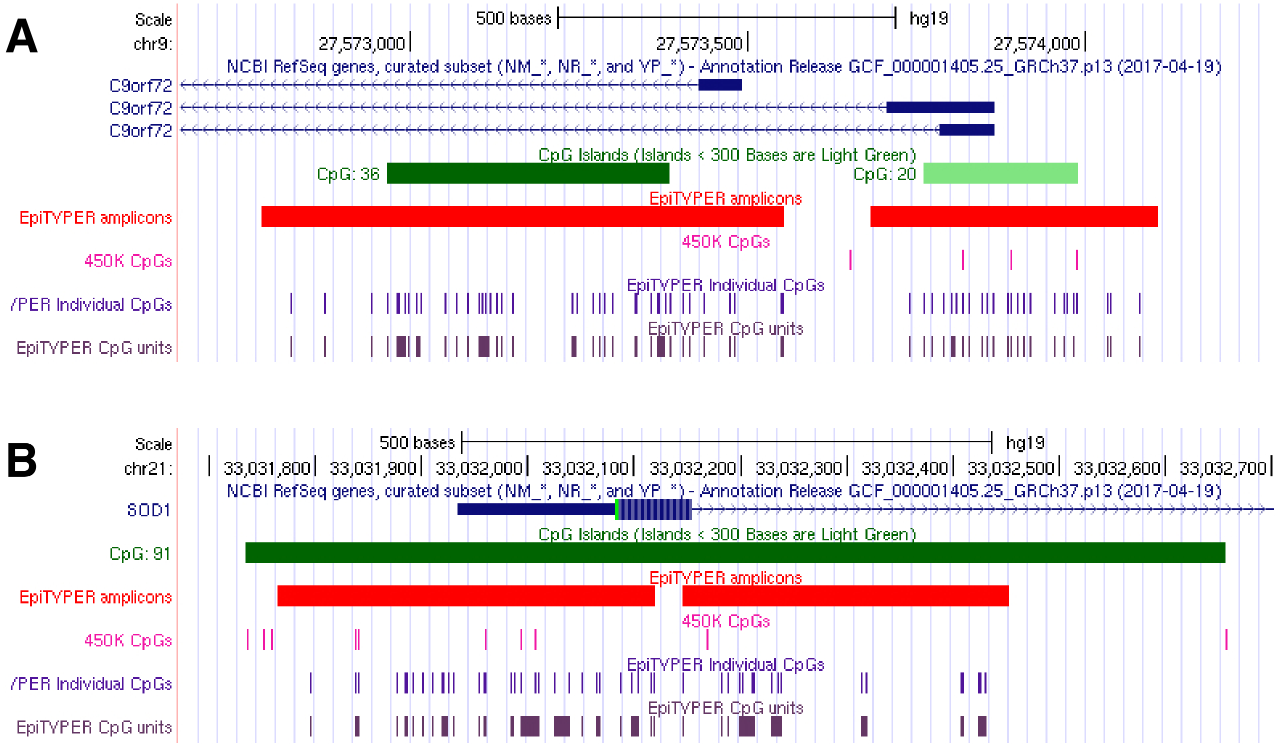

Supplementary Figure 1. EpiTYPER CpG units and 450K CpG probe coverage of *SOD1* and *C9orf72* CpG islands.
Methylation of the promoter regions of *C9orf72* (**A**) and *SOD1* (**B**) were assayed using both custom EpiTYPER amplicons and 450K CpG probes. Amplicons and probes are shown in relation to CpG islands and gene features.

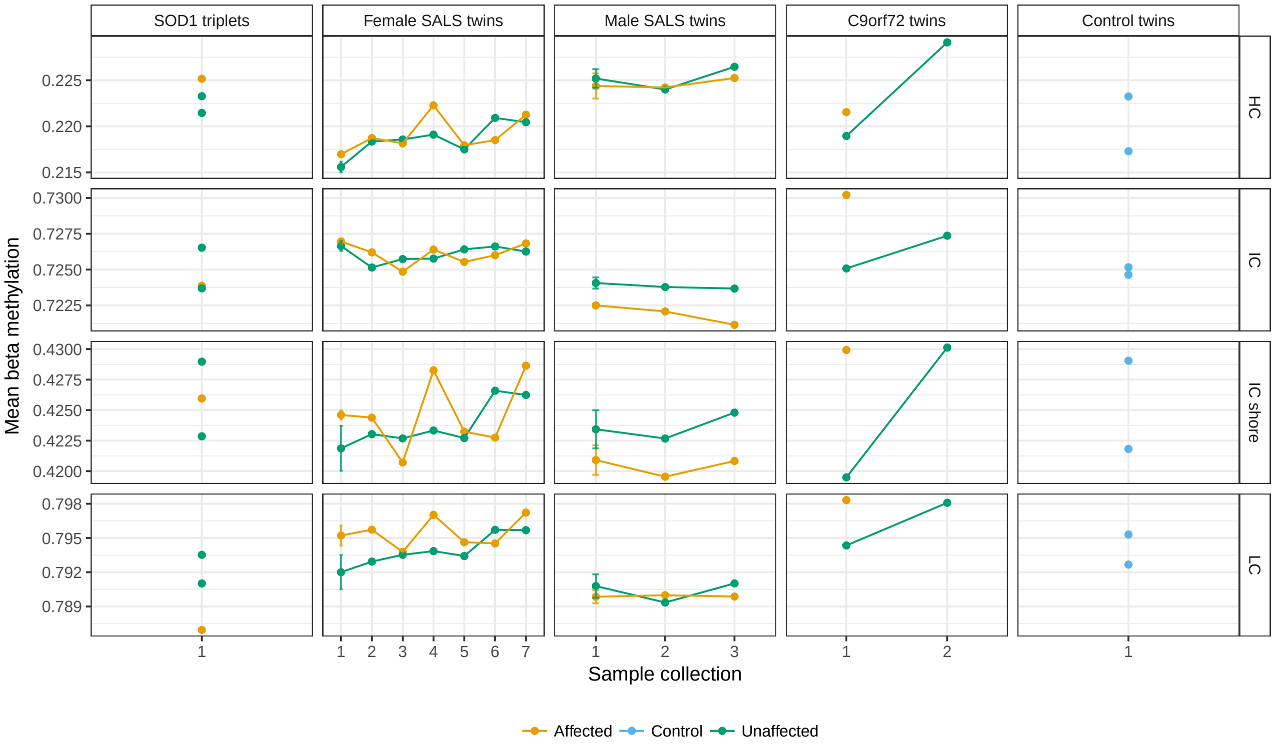

Supplementary Figure 2. Mean methylation does not differ by disease status within HIL classes. 386,183 CpGs were separated according to HIL class and mean methylation calculated across all 133192 high density CpG island probes (HC), 88,286 intermediate density CpG island probes (IC), 28,661 intermediate density CpG island shore probes, and 136044 low density non-island probes. Mean methylation over time is shown for all twin/triplet sets. Mean methylation did not vary between ALS-affected and unaffected co-twins/-triplets in any class (HC, $p = 0.93$; IC, $p = 0.99$; ICshore, $p = 0.82$; LC, $p = 0.093$) when controlling for age and sex in a mixed model.

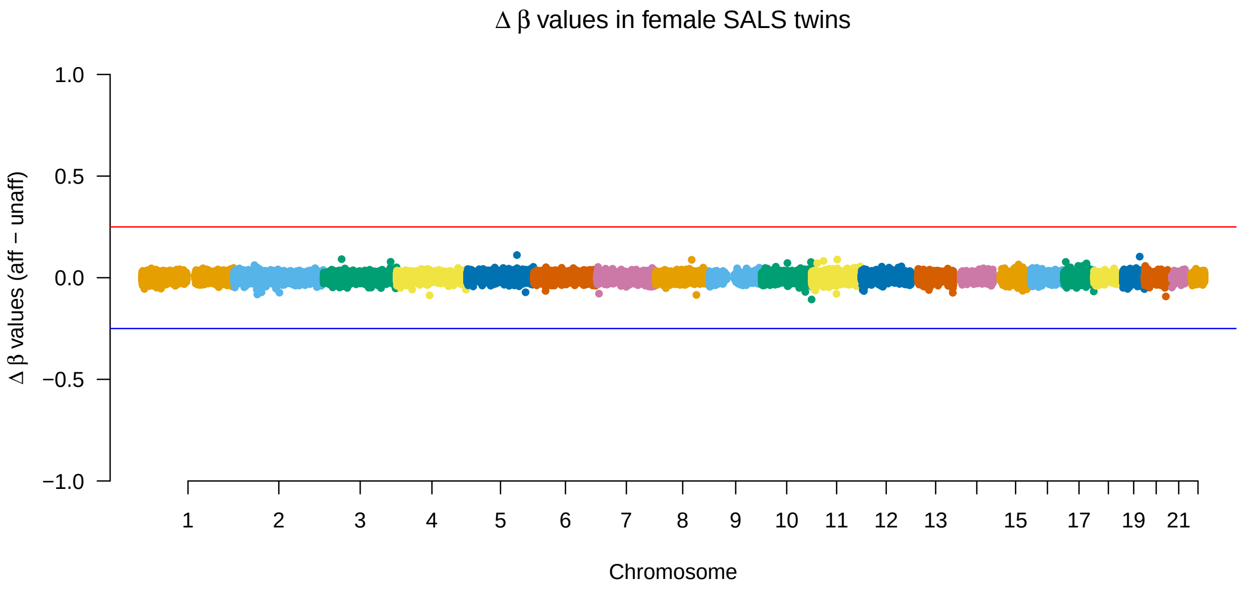

Supplementary Figure 3A. Using a $\Delta\beta$-methylation ≥ 0.25 threshold, we identified no differentially methylated probes in the female SALS twins

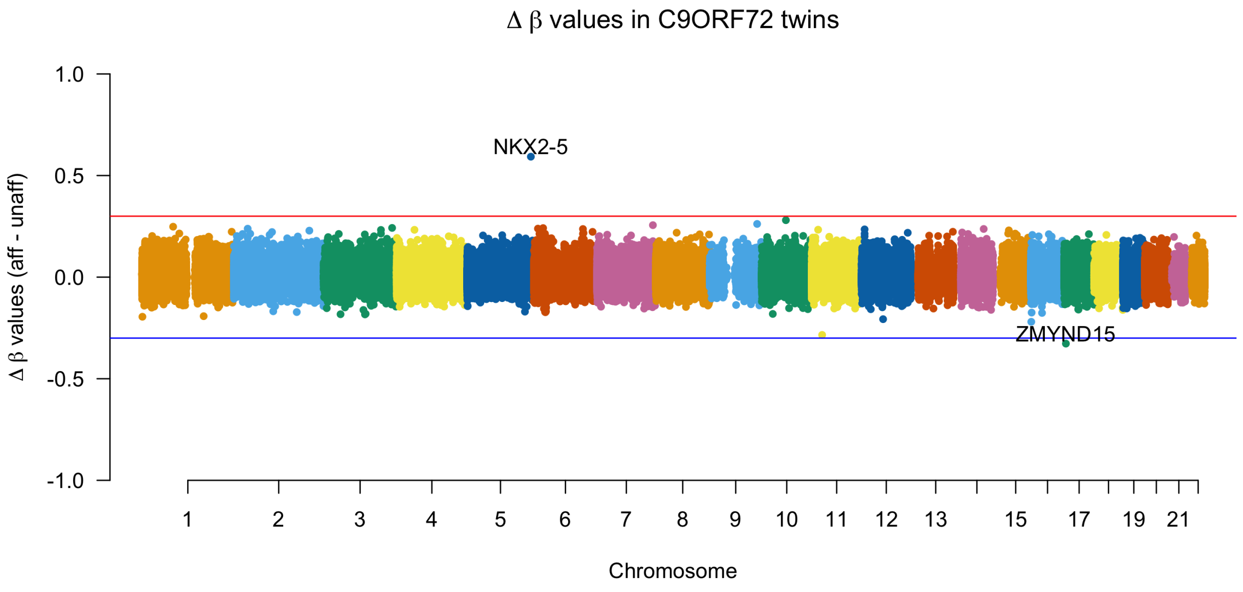

Supplementary Figure 3B. Using a $\Delta\beta$-methylation ≥ 0.25 threshold, we identified 6 differentially methylated probes in the *C9orf72* twins. Probes above the red line are hypermethylated in the affected co-twin whereas probes below the blue line are hypermethylated in the unaffected co-twin (therefore hypomethylated in the affected co-twin.

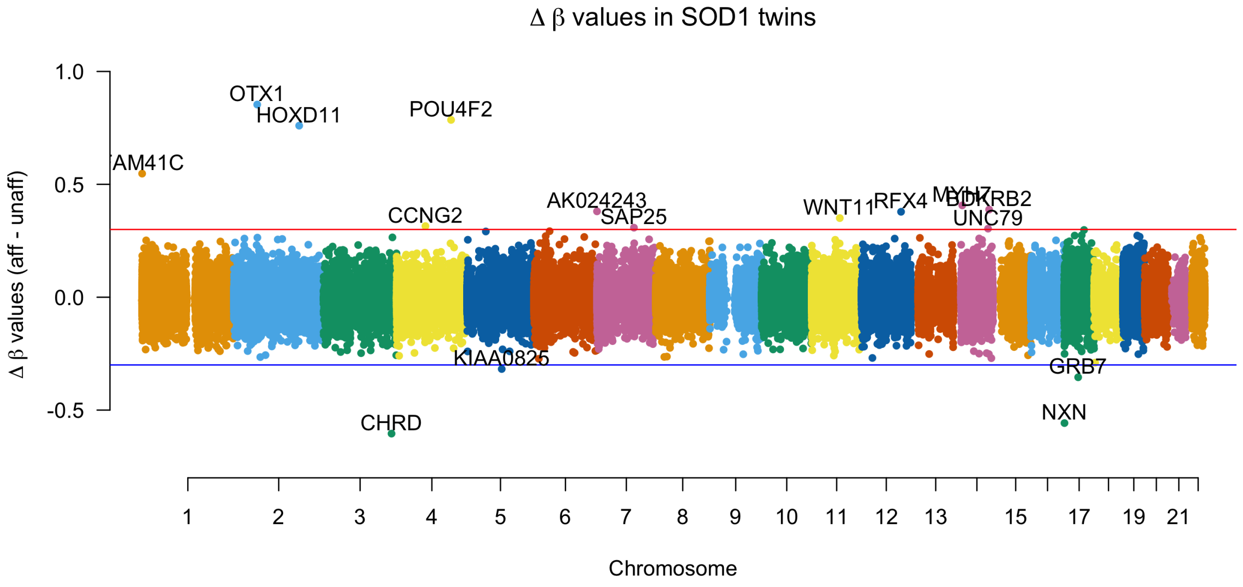

Supplementary Figure 3C. Using a Δβ-methylation $\geq$ 0.25 threshold, we identified 58 differentially methylated probes in the *SOD1* triplets. Probes above the red line are hypermethylated in the affected co-triplet whereas probes below the blue line are hypomethylated in the affected co-triplet.

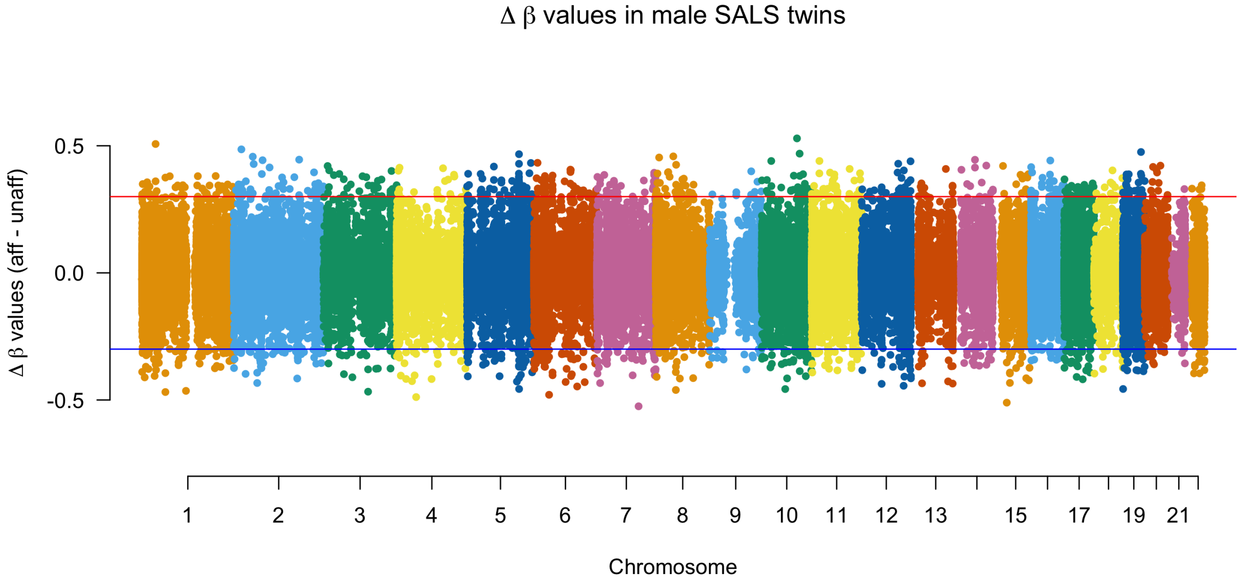

Supplementary Figure 3D. Using a Δβ-methylation $\geq$ 0.25 threshold, we identified 2,689 differentially methylated probes in the male SALS twins

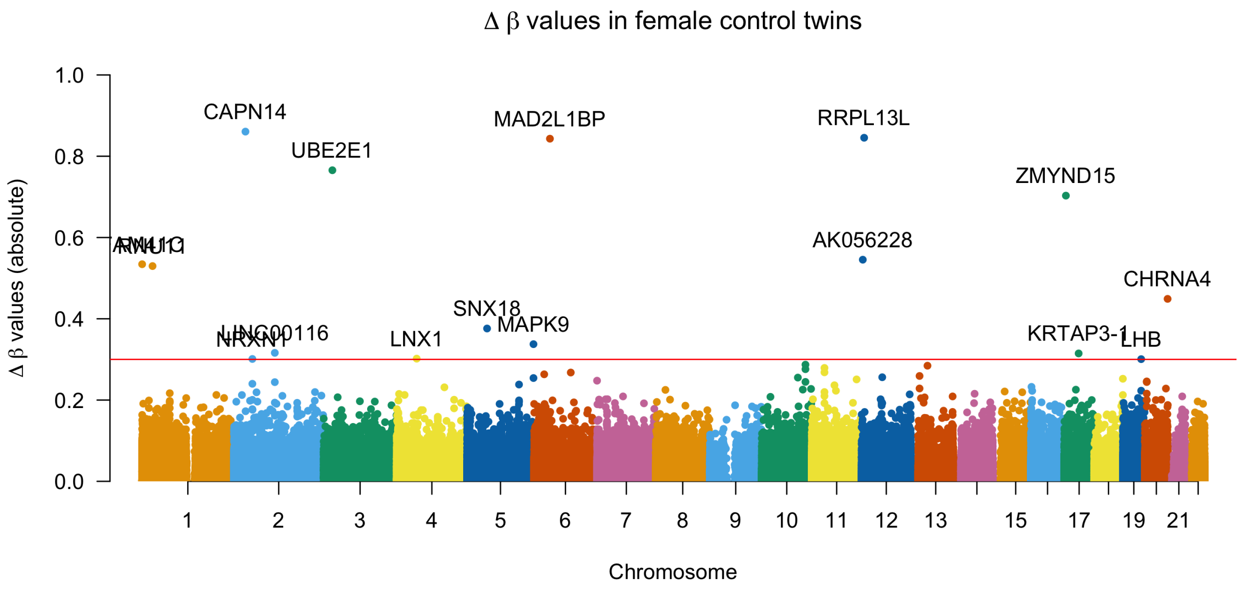

Supplementary Figure 3E. Using a $\Delta\beta$-methylation ≥ 0.25 threshold, we identified 29 differentially methylated probes in the control twins
